# Effects of intrafractional diaphragm motion on dose perturbation in stereotactic body radiation therapy for lower thoracic vertebrae

**DOI:** 10.1016/j.phro.2025.100780

**Published:** 2025-05-13

**Authors:** Fumiyasu Matsubayashi, Kosuke Matsuura, Yasushi Ito, Yasuo Yoshioka

**Affiliations:** Radiation Oncology Department, Cancer Institute Hospital, Japanese Foundation for Cancer Research, 3-8-31 Ariake, Koto-ku, Tokyo 135-8550, Japan

**Keywords:** Stereotactic body radiation therapy, Spinal metastases, Volumetric-modulated arc therapy, Intrafractional diaphragm motion, Dose perturbation

## Abstract

**Background and Purpose:**

This study aimed to evaluate the impact of intrafractional diaphragm motion (IFDM) on dose accuracy in stereotactic body radiation therapy (SBRT) for lower thoracic vertebrae.

**Materials and Methods:**

A retrospective analysis was conducted on 10 patients who underwent SBRT using volumetric-modulated arc therapy (SBRT-VMAT) for the lower thoracic vertebrae. For all patients, dynamic dose calculation (DDC) was performed, incorporating IFDM using arc-divided VMAT plans, respiratory waveforms, and four-dimensional computed tomography (4DCT). The DDC results were compared with doses calculated using time-averaging CT (AveCT) and individual-phase CT scans. Diaphragm motion was quantified using 4DCT, and the correlation between IFDM and dose perturbation was assessed.

**Results:**

The minimum gross tumor volume (GTV) dose was overestimated by 1.8 % in phase 0 % and underestimated by − 1.0 % in phase 50 %. A statistically significant correlation was observed between dose variation and the magnitude of IFDM. In the case with the greatest magnitude of diaphragm motion, a 4.3 % variation in GTV was observed compared with the DDC. By contrast, mid-ventilation CT and AveCT showed a mean dose variation of < 0.7 %.

**Conclusion:**

This study incorporated IFDM into dose calculation for SBRT-VMAT. Static planning based on CT scans acquired at a specific phase may result in unexpected dose variations. Mid-ventilation CT and AveCT demonstrated utility in mitigating dose variations associated with IFDM. Considering the correlation between dose variation and diaphragm motion magnitude is crucial for developing effective dose perturbation strategies for IFDM.

## Highlights

• The doses considering diaphragm motion during spine irradiation were assessed.
• A 4.3% variation in target dose due to diaphragm motion (DM) was observed.
• Improper planning commuted tomography (CT) caused dose variation from DM.
• Dose variation was predicted using correlations with DM.
• Time-averaged CT provided the most robust planning dose for accuracy.

## 1 Introduction

Stereotactic body radiation therapy (SBRT) is a technique that delivers high biologically effective doses to targets while minimizing doses to normal tissue over a few fractions [1]. For spinal metastases, SBRT has emerged as an alternative to conventional palliative radiation therapy and has shown superior effectiveness in pain relief compared with conventional radiation therapy [2]. In most cases, the target surrounds the spinal cord, necessitating the use of intensity-modulated radiation therapy or volumetric-modulated arc therapy (VMAT) in spine SBRT [3].

When performing SBRT to the lower thoracic vertebrae, doses to the target and spinal cord can be influenced by respiratory motion of the diaphragm, which alters the water-equivalent beam path despite the fact that the target and spinal cord remain static. Wang et al. [4] evaluated dose variations in the target and spinal cord caused by breathing by calculating doses using four-dimensional computed tomography (4DCT). Their findings revealed that dose coverage in the gross tumor volume (GTV) could vary by up to 6.5 % in the worst-case scenario. Similarly, Okamoto et al. [5] investigated the dosimetric impact of liver movement using inhale and exhale computed tomography (CT) and proposed a gantry angle selection strategy in SBRT using VMAT (SBRT-VMAT) to mitigate this impact. These studies highlighted dose variations resulting from diaphragm displacement. However, during free-breathing treatments, the diaphragm moves continuously due to respiration while VMAT irradiation is delivered. As a result, these studies do not fully account for intrafractional diaphragm motion (IFDM) during treatment. IFDM is a crucial factor because it can cause dose variations due to interplay effects [6,7]. The purpose of this study was to perform dynamic dose calculations (DDCs) accounting for IFDM in SBRT-VMAT for the lower thoracic vertebrae and to evaluate the impact of IFDM on dose calculation accuracy.

## 2 Materials and methods

### 2.1 Patient characteristics

We conducted a retrospective analysis of patients who underwent SBRT-VMAT for spinal metastases at our institution between 2020 and 2024. Patients were included if the metastatic site was located in the 7th through 12th thoracic vertebrae. Ten patients were ultimately included in this study. The patients’ characteristics and treatment details are summarized in Supplementary Table S1. This study was approved by the institutional review board of our institution (review board number: 2024-GB-026).

### 2.2 CT simulation and treatment planning

All patients were positioned supine with their arms raised. A custom-molded vacuum cushion (Vac-Lok; CQ Medical, Avondale, PA, USA) was used for each patient to enhance setup reproducibility and comfort. Additionally, a thermoplastic body shell (HipFix thermoplastic positioning system; CQ Medical) was applied to the thoraco-abdominal region to stabilize the posture and reduce deep breathing by compressing the abdomen.

For CT acquisition, surrogate respiratory waveforms were recorded using the Respiratory Gating for Scanners (RGSC) system (Varian Medical Systems, Palo Alto, CA, USA) for 4DCT. The 4DCT images were acquired using a SOMATOM Confidence RT Pro scanner (Siemens Healthineers, Erlangen, Germany) and divided into 10 image sets corresponding to respiratory phases (0 %–90 %, with 0 % representing peak inspiration and 50 % representing end expiration) based on the respiratory cycle. Additionally, a time-averaged CT (AveCT) scan was reconstructed using CT data from all phases.

Magnetic resonance imaging (MRI) acquired prior to treatment planning, with the patient immobilized, was fused with AveCT using bony rigid registration. The target and organs at risk were delineated on AveCT with reference to the registered MRI. Gross disease was contoured as the GTV, and the clinical target volume (CTV) was contoured following consensus contouring guidelines [8]. A margin of 0–2 mm was added to the CTV to define the planning target volume (PTV). The spinal cord was contoured with reference to the fused MRI. All contours were then copied to each phase-specific CT scan using bony-based rigid registration.

Treatment planning was performed using Eclipse version 16.1 (Varian Medical Systems). The VMAT irradiation technique utilized two or four full arcs and a 10-MV flattening-filter-free beam. Treatment planning and dose calculation were performed using AveCT. The plan was then copied onto each phase-specific CT scan, and doses were recomputed using the AcurosXB algorithm. The prescribed dose for each patient is shown in Supplementary Table S1.

### 2.3 Quantification of diaphragm motion and anatomical changes

Diaphragm motion was quantified using 4DCT, and anatomical changes were examined based on CT values. The body area at the CT slice corresponding to the center of the GTV in phase *p* (*D*_body,_*_p_*) was measured. Similarly, the lung area at the same CT slice in the 0 % and 50 % phase CT scans (*D*_lung0%_, *D*_lung50%_, respectively) was measured. The diaphragm motion indicator (*ΔD*_dia_) for each patient was then calculated using Equation (1):

(1) $$\Delta D_{dia}=\frac{D_{lung0\%}-D_{lung50\%}}{D_{body,\;0\%}}\left(\%\right)$$

Anatomical change was assessed by evaluating variations in mean CT values of the body. CT values in *D*_body,_*_p_* were measured for each patient (*n*) as *S_p,n_*, and changes in CT values (*ΔHU_p_*) due to phase variations relative to the 20 % phase (mid-position of the diaphragm) were calculated using Equation (2):

(2) $${\Delta HU}_{p}=\frac{\sum_{n=1}^{10}S_{p,n}-\sum_{n=1}^{10}S_{20\%,n}}{10}$$

### 2.4 DDC accounting for IFDM

Supplementary Fig. S1 shows a schematic diagram of the DDC implementation. Sub-arcs were generated by dividing a VMAT plan into 24 arcs per full arc (with each sub-arc spanning 15 degrees of rotation), resulting in a total of 48–96 sub-arcs per plan. The midpoint of the time duration for each sub-arc was calculated based on the plan parameters. Similarly, the respiratory phase duration was determined using the patient’s respiratory period and the time duration, assuming that arc irradiation began at phase 0 %, 25 %, and 50 %. The respiratory phase for each sub-arc was calculated based on the respiratory phase duration, the midpoint time of each sub-arc, and the irradiation start phase. Dose calculations were performed for each phase-specific CT scan using only the sub-arcs corresponding to the respective phases in the 4DCT. Finally, 30 dose distributions were generated, accounting for the 3 irradiation start phases across the 10 phase-specific CT scans. These distributions were then summed onto AveCT using the bony registration matrix derived from each CT image.

### 2.5 Dose evaluation

We evaluated dose variations in static plans for each phase CT and AveCT compared with the DDC plans. The dose indices (DIs) assessed included the minimum dose for the GTV (GTV_min_), the dose covering 90 % of the CTV volume (CTV_D90%_), and the dose covering 0.01 cc of the spinal cord (Cord_0.01cc_). The variations of each DI in phase *p* for patient *n* (*ΔDI_p_*_,_*_n_*) was calculated using Equation (3):

(3) $${\Delta DI}_{p,n}=\;\frac{{\mathrm{DI}}_{p,n}-{\mathrm{DI}}_{DDC,n}}{{\mathrm{DI}}_{DDC,n}}$$

The mean and standard deviation of *ΔDI_p_,_n_* in phase *p* (*M_p_* and *SD_p_*, respectively) were calculated using Eqs. (4), (5):

(4) $$M_{p}=\frac{\sum_{n=1}^{10}{\Delta DI}_{p,n}}{10}$$

(5) $${\mathrm{SD}}_{p}=\sqrt{\frac{\sum_{n=1}^{10}\left({\Delta DI}_{p,n}-M_{p}\right)}{10}}$$

Additionally, we examined the correlation between each *ΔDI_p_,_n_* and *ΔD*_dia_, as well as *M_p_* and *ΔHU_p_*, by Spearman’s rank correlation test using EZR [9].

## 3 Results

**Fig. 1:**
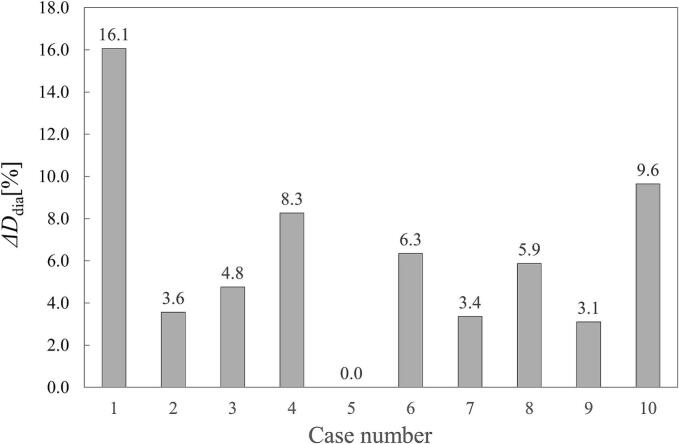
Diaphragm motion indicator (*ΔD*_dia_) for each case.

The diaphragm motion indicator (*ΔD*_dia_) for each patient is shown in Fig. 1. The mean *ΔD*_dia_ was 6.1 %, with Case #1 (site: T10) exhibiting the largest *ΔD*_dia_ of 16.1 %. Case #5 (site: T11) showed no volume change in the diaphragm within the CT slice at the GTV center, despite observed lung expansion and contraction during breathing. Changes in the mean body CT values (*ΔHU_p_*) are illustrated in Supplementary Fig. S2 Δ*HU_p_* decreased during inspiration (near the 0 % phase) and increased during expiration (near the 50 % phase). The difference in *ΔHU_p_* between the 0 % and 50 % phases was 60 HU.

**Fig. 2:**
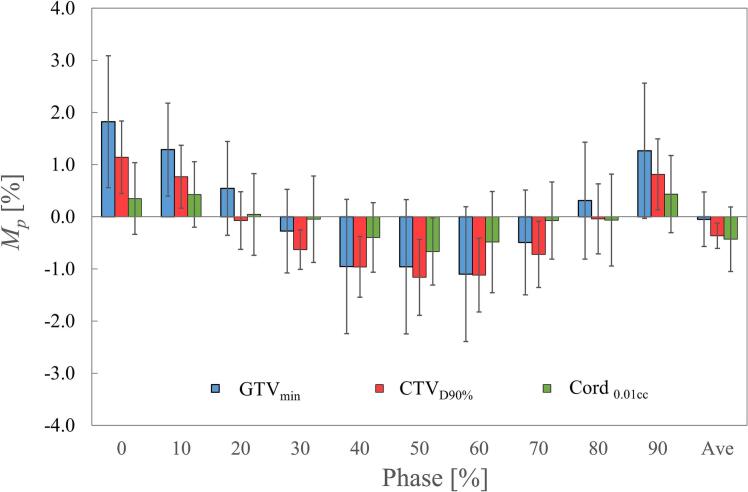
Mean dose variation (*M_p_*) and standard deviation (*SD_p_*) for each phase. *M_p_* includes the minimum dose for GTV (GTV_min_), the dose covering 90 % of the CTV volume (CTV_D90%_), and the dose covering 0.01 cc for the spinal cord (Cord_0.01cc_). Error bars indicate *SD_p_* in each phase.

**Fig. 3:**
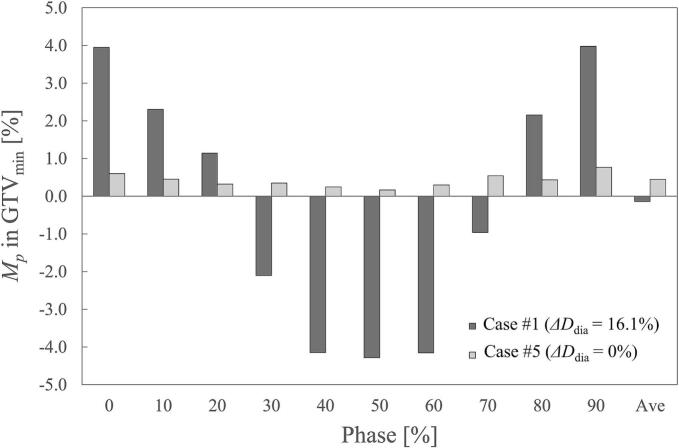
Variation of *M_p_* in the minimum dose for GTV (GTV_min_) in Cases #1 and #5 across phases. Case #1 exhibited the largest *ΔD*_dia_ (16.1%), while no diaphragm volume change was observed in Case #5.

The *M_p_* and *SD_p_* for each DI are presented in Fig. 2. Regarding *M_p_*, phase 0 % showed an overestimation of 1.8 %, 1.1 %, and 0.4 % for GTV_min_, CTV_D90%_, and Cord_0.01cc_, respectively. Conversely, phase 50 % exhibited an underestimation of − 1.0 % for GTV_min_, −1.2 % for CTV_D90%_, and − 0.7 % for Cord_0.01cc_. By contrast, all *M_p_* values in the mid-position phases (20 %–30 % and 70 %–80 %) and in AveCT were within 0.7 %. The *SD_p_* values (show by error bars in Fig. 2) remained relatively constant across phases 0 %–90 %, with mean values of 1.1 % for GTV_min_, 0.6 % for CTV_D90%_, and 0.8 % for Cord_0.01cc_. Among all phase-specific CT scans, AveCT exhibited the smallest *SD_p_*. Fig. 3 illustrates the *M_p_* values in GTV_min_ for Case #1, which had the largest *ΔD*_dia_, and Case #5, which exhibited no diaphragm volume change. For Case #1, the variation between phase 0 % and phase 50 % was 8.2 %; however, AveCT exhibited a dose variation of < 0.2 % compared with the DDC plan, even in this extreme case. In Case #5, the maximum *M_p_* variation was 0.7 %.

**Fig. 4:**
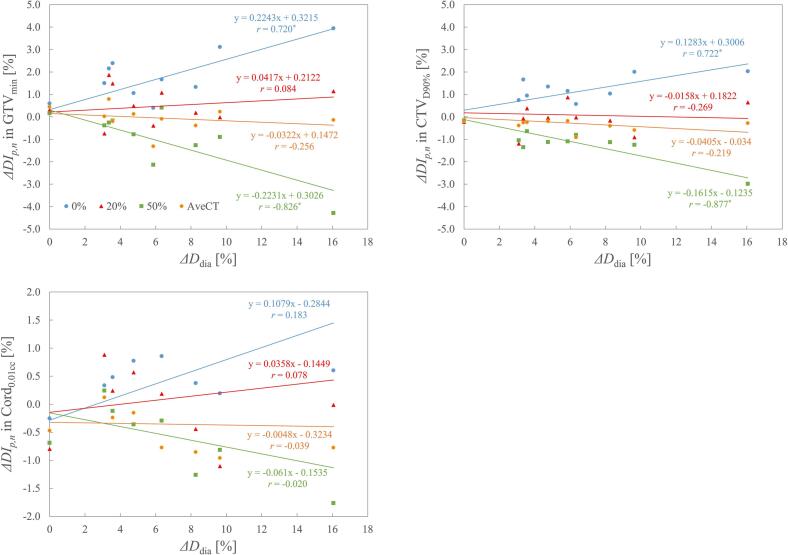
Relationship between variation in *ΔDI_p_,_n_* and *ΔD*_dia_ for phases 0 %, 20 %, 50 %, and time-averaging CT (AveCT). Linear approximation results are shown, along with the corresponding approximation formulas and correlation coefficients. Statistically significant correlation coefficients (p < 0.05) are marked with asterisks (*).

**Fig. 5:**
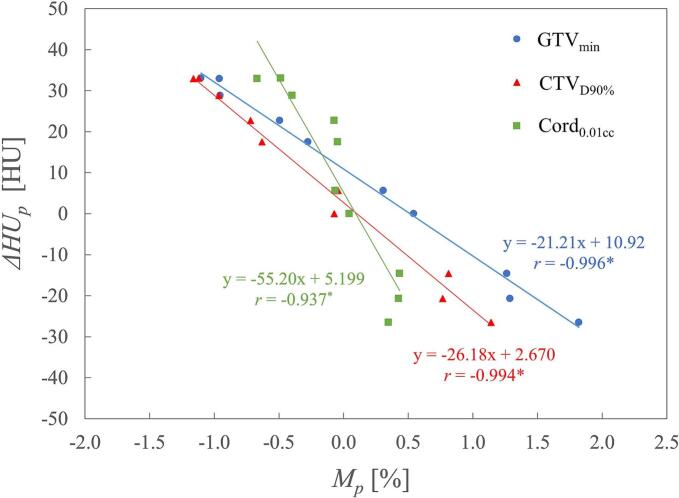
Relationship between *M_p_* in each DI and *ΔHU_p_*. Linear approximation results are shown, along with the corresponding approximation formulas and correlation coefficients. Statistically significant correlation coefficients (p < 0.05) are marked with asterisks (*).

The relationship between *ΔDI_p_,_n_* and *ΔD*_dia_ for phases 0 %, 50 %, 20 % (mid-ventilation), and AveCT is shown in Fig. 4. In phase 0 %, *ΔDI_p_,_n_* tended to increase with larger *ΔD*_dia_, whereas in phase 50 %, it tended to decrease. A statistically significant correlation was observed in phases 0 % and 50 % for GTV_min_ and CTV_D90%_. Regardless of the variations in *ΔD*_dia_, phase 20 % and AveCT demonstrated relatively low and stable variations. The relationship between *M_p_* and *ΔHU_p_* is shown in Fig. 5. *ΔHU_p_* tended to decrease as *M_p_* increased, showing a significant correlation between the linear approximation and the data for all DIs. A 1 % increase in *M_p_* corresponded to a decrease in *ΔHU_p_* of 21 HU for GTV_min_, 26 HU for CTV_D90%_, and 55 HU for Cord_0.01cc_.

## 4 Discussion

This study investigated the impact of IFDM on dose accuracy in SBRT-VMAT for the lower thoracic vertebrae. The results demonstrated the potential for dose variations when IFDM is not accounted for in treatment planning, particularly in cases with significant diaphragm motion. A correlation between dose variation and the diaphragm motion indicator (*ΔD*_dia_) was found in GTV_min_ and CTV_D90%_. Deformable image registration is often used for dose summation and displacement measurement because moving targets cause geometric inconsistency [10,11]. For dose summation, previous studies have shown that thoracic vertebral motion during respiration is within 0.1 mm [4] and does not involve deformation. Based on these findings, we summed doses in DDC using rigid bony registration. Regarding displacement measurement, this approach includes information outside the PTV’s CT plane. By contrast, our motion indicator specifically measures diaphragm motion within this plane, aligning with surrogate measurement methods used in prior research [5,12].

Compared with DDC, *M_p_* in the mid-position phases (20 %–30 % and 70 %–80 %) and in AveCT agreed within 1 %. However, *M_p_* was overestimated at the 0 % phase and underestimated at the 50 % phase. This variation likely resulted from changes in the water-equivalent length in each phase-specific CT scan. A previous study reported that a ± 30 HU change in soft tissue can affect the dose by approximately ± 1 % [13]. Our findings (Fig. 5) demonstrate a negative correlation between *M_p_* and *ΔHU_p_*, with dose change slopes closely matching previously reported values (21 HU for GTV_min_, 26 HU for CTV_D90%_, and 55 HU for Cord_0.01cc_ per 1 % *M_p_* variation). Consequently, lung expansion due to inspiration reduces the water-equivalent length at the 0 % phase, resulting in a dose increase. By contrast, at the 50 % phase, an increase in water-equivalent length was observed, leading to a dose decrease. In terms of *M_p_* variations, the mid-position phases and AveCT can effectively represent water-equivalent length in the presence of IFDM.

In SBRT for lung tumors, it has been reported that the interplay effect between the multi-leaf collimator and tumor motion has no impact on GTV dose because of the averaging effect [14,15]. However, in our study, despite averaging three DDC plans with varying irradiation start phases, some cases—particularly those with large *ΔD*_dia_—still exhibited dose variations of approximately 4 % between the averaged DDC plan and static plans using phase-specific CT scans. The dose variation caused by discrepancies in IFDM representation between the treatment planning CT and actual treatment delivery is not averaged out over fractions. Therefore, if IFDM is not accounted for in the planning process, systematic dose variations between the planned and actual dose may occur. Previous studies have shown that expanding the PTV margin reduces dose errors caused by the interplay effect in SBRT for lung tumors [16]. However, PTV margins are typically added to compensate for dose coverage affected by target motion; therefore, such expansion may be ineffective in this case because IFDM occurs outside the PTV. Unfortunately, no clear consensus exists on this issue.

In cases with a large *ΔD*_dia_, such as Case #1, treatment planning based on free-breathing CT with an unknown respiratory phase may introduce uncertainties caused by IFDM, reaching up to ± 4 % in GTV. The doses calculated using AveCT or mid-ventilation CT (phase 20 %–30 % or 70 %–80 %) showed relatively good agreement with the DDC dose. Considering dose certainty, as shown by the standard deviations in Fig. 2, AveCT was the most suitable representation of the DDC dose in static dose calculations.

Regarding the correlation between *ΔDI_p_,_n_* and *ΔD*_dia_, statistically significant correlations were observed in phases 0 % and 50 % for all DIs except for the spinal cord. By contrast, *ΔDI_p_,_n_* in AveCT and phase 20 % remained consistently low, regardless of changes in *ΔD*_dia_. Because the spinal cord is frequently shielded by the multi-leaf collimator in spinal SBRT-VMAT, it is less affected by IFDM than is the target. Consequently, *ΔDI_p_,_n_* for Cord_0.01cc_ showed no correlation with *ΔD*_dia_ in all DIs. According to the approximate formulas for the correlation, *ΔDI_p_,_n_* for GTV_min_ can be estimated to change by approximately 0.2 % (averaging phases 0 % and 50 %) for every 1 % change in *ΔD*_dia_. Similarly, *ΔDI_p_,_n_* for CTV_D90%_ is estimated to change by approximately 0.2 % for every 1 % change in *ΔD*_dia_. Using these formulas, it is possible to predict dose variation due to IFDM based on *ΔD*_dia_ calculated from a 4DCT or inhalation-exhalation CT prior to treatment. This prediction can guide the selection of an IFDM dose perturbation strategy. For instance, free-breathing CT can be used for treatment planning in cases with low *ΔD*_dia_, while AveCT may be appropriate for cases with unacceptable dose variation when using free-breathing CT. Alternatively, gated-CT acquisition in mid-ventilation can be utilized for free-breathing irradiation to mitigate dose variation. It is important to recognize that even in cases with *ΔD*_dia_ of 0 %, a dose variation of approximately 0.7 % was observed. This uncertainty likely affects all patients and appears to stem from 4DCT accuracy issues, such as the correctness of volume representation [17] and 4DCT-specific artifacts [18]. Therefore, an inherent level of uncertainty must be considered when performing DDC.

This study has some limitations. Because of the small sample size and variations in prescribed dose, we cannot rule out the possibility of bias in our results. Further investigation with a larger sample size is necessary. However, despite the limited number of cases in this study, statistically significant correlations were observed in some of the data, suggesting that our findings are meaningful. Additionally, considering the clinically required accuracy (±5%) [19], the variations observed in this study do not represent clinically significant deviations. Furthermore, other uncertainties exist, including those related to the dose calculation algorithm [20], dose reporting [21], and inter- or intra-fractional setup errors [22,23]. The reproducibility of respiration between 4DCT and treatment delivery is also a crucial concern. However, previous studies have performed DDC in lung SBRT using 4D cone-beam CT acquired during online treatment [24,25]. If this technique could be applied to IFDM, it would allow for real-time incorporation of respiratory motion into DDC. To mitigate potential issues during irradiation, continuous monitoring of the patient’s respiration using a surrogate respiratory monitor is essential. However, it is worth noting that while all treatment delivery plans in our study met clinical goals, one DDC plan did not. Thus, failing to account for IFDM poses a potential risk. It is important to consider the impact of IFDM on dose calculation accuracy when performing SBRT-VMAT for the lower thoracic vertebrae. In conclusion, we performed DDCs accounting for IFDM in SBRT-VMAT for the lower thoracic vertebrae and assessed the accuracy of dose calculations on phase CT and AveCT by comparing them with the DDCs. The results demonstrated that using an inappropriate phase CT for dose calculation could lead to significant discrepancies between the calculated and actual doses. We propose that pre-measurement of diaphragm motion can be utilized to predict these discrepancies. Among the approaches evaluated, AveCT proved to be the most robust and reliable method for dose calculation when compared with DDC.

## CRediT authorship contribution statement

**Fumiyasu Matsubayashi:** Conceptualization, Methodology, Validation, Investigation, Writing – original draft. **Kosuke Matsuura:** Investigation. **Yasushi Ito:** Methodology, Investigation. **Yasuo Yoshioka:** Writing – review & editing, Supervision.

## Funding

This study was supported by an institutional research budget provided by the Japanese Foundation for Cancer Research.

## Declaration of competing interest

The authors declare that they have no known competing financial interests or personal relationships that could have appeared to influence the work reported in this paper.
