# Supplementary material for: Effects of intrafractional diaphragm motion on dose perturbation in stereotactic body radiation therapy for lower thoracic vertebrae
Source: Phys Imaging Radiat Oncol. 2025 May 13;34:100780. doi: 10.1016/j.phro.2025.100780 (PMC12145723; doi:10.1016/j.phro.2025.100780)
Supplement: Supplementary Data 1 [file mmc1.pdf]

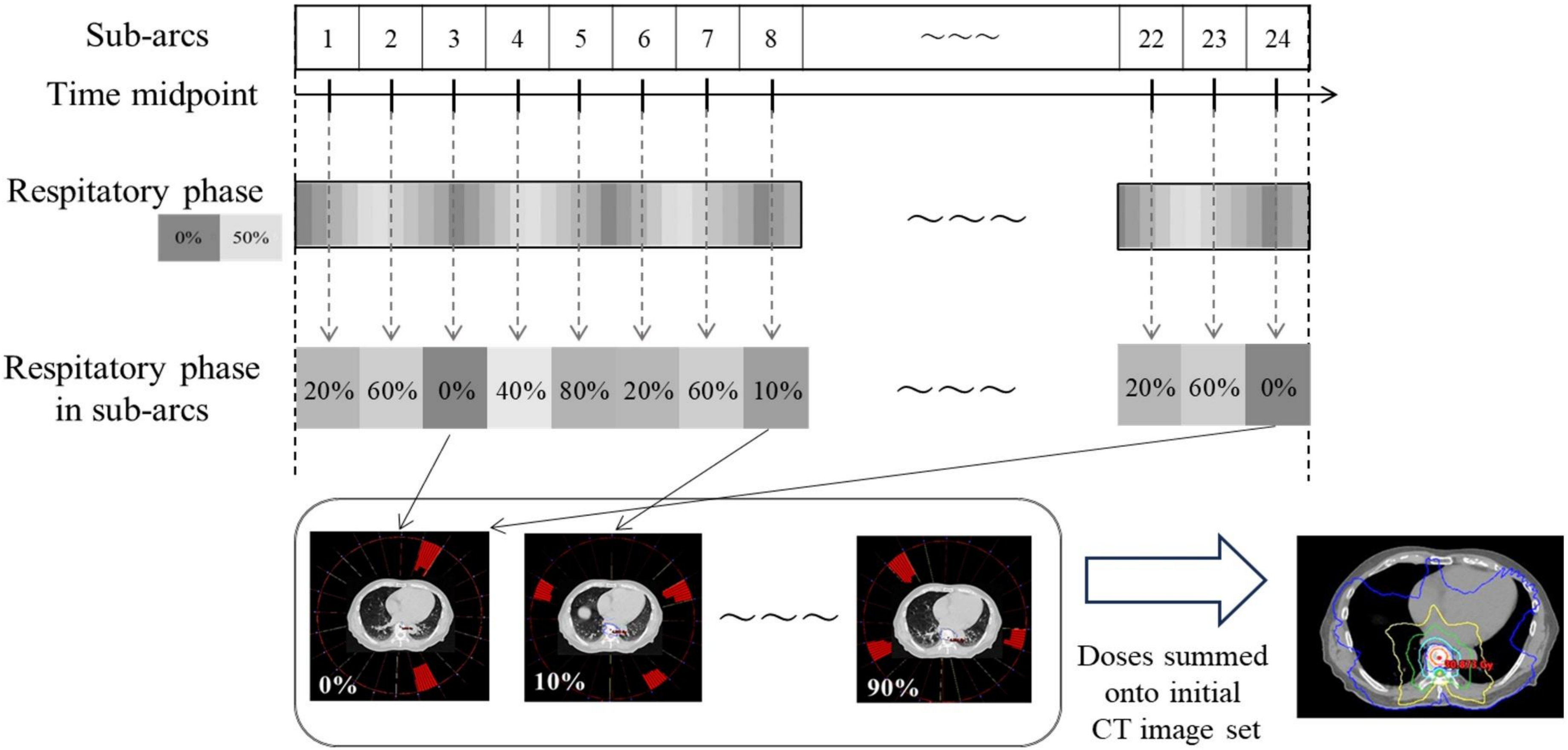

Fig. S1 Schematic diagram illustrating the implementation of the dynamic dose calculation (DDC).

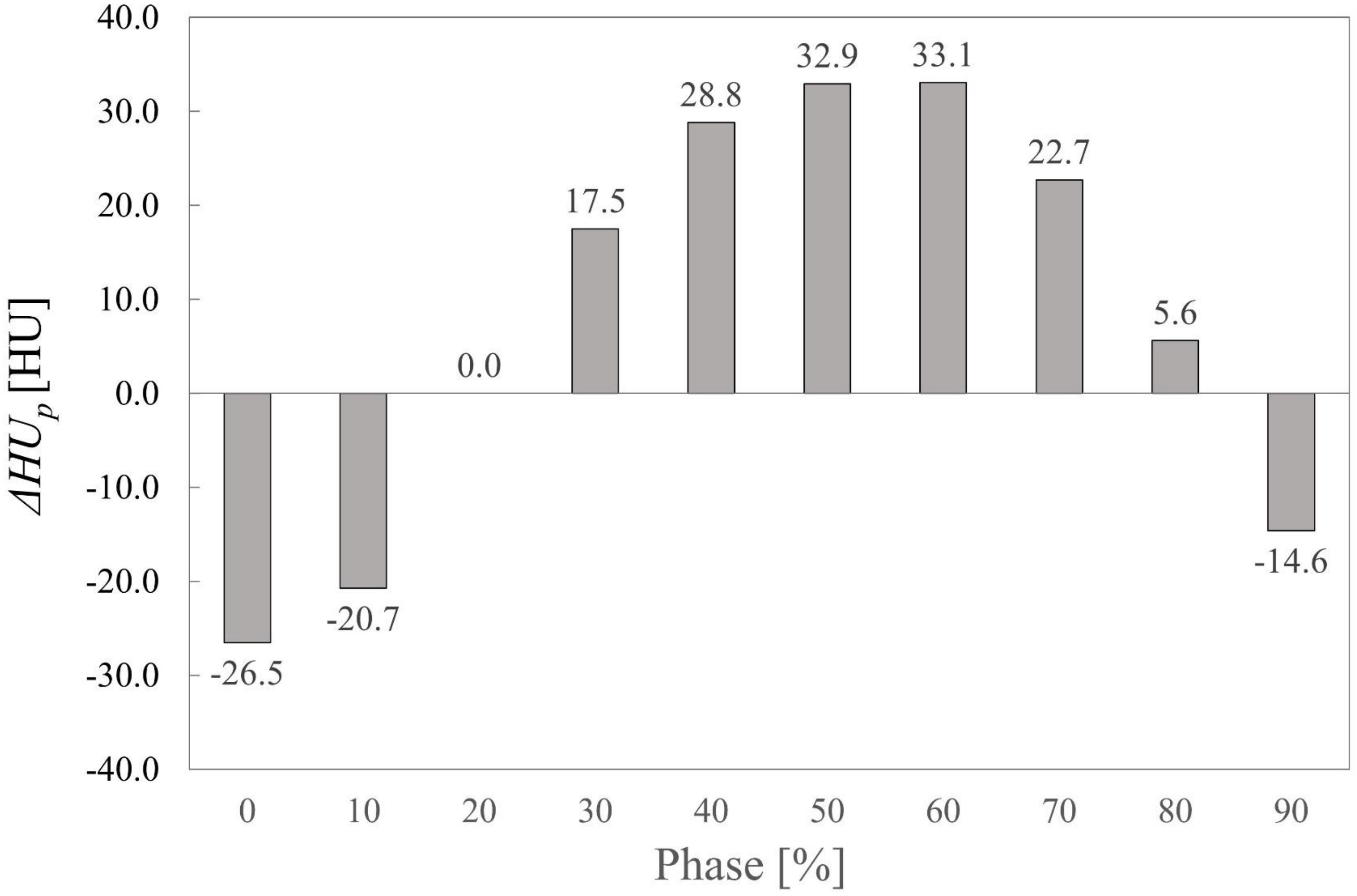

Fig. S2 Changes in the mean CT value ($\mathit{\Delta HU_p}$) of the body contour for each phase. $\mathit{\Delta HU_p}$ represents the difference in the mean CT value relative to the 20% phase.

Table S1 Patient characteristics and treatment information

Case number	Site	Irradiation history (yes or no)	D_{min} to gross tomor volume	$D_{90\%}$ to clinical target volume	Fractions	Respiratory period
1	T10	No	24 Gy	16 Gy	1	3.8 seconds
2	T9	Yes	27 Gy	21 Gy	3	3.4 seconds
3	T10	No	30 Gy	27 Gy	5	3.4 seconds
4	T10	No	27 Gy	21 Gy	3	4.1 seconds
5	T11	No	27 Gy	21 Gy	3	3.2 seconds
6	T10	No	27 Gy	24 Gy	3	6.4 seconds
7	T8	No	27 Gy	21 Gy	3	3.1 seconds
8	T9	Yes	30 Gy	25 Gy	5	4.0 seconds
9	T10	No	27 Gy	24 Gy	3	3.0 seconds
10	T11	Yes	25 Gy	24 Gy	5	5.9 seconds

D_{min} : minimum dose, $D_{90\%}$: doses covering 90% of the volume
